# Design Methodology of Microservices to Support Predictive Analytics for IoT Applications

**DOI:** 10.3390/s18124226

**Published:** 2018-12-02

**Authors:** Sajjad Ali, Muhammad Aslam Jarwar, Ilyoung Chong

**Affiliations:** Department of Information and Communications Engineering, Hankuk University of Foreign Studies, Seoul 02450, Korea; sajjad@hufs.ac.kr (S.A.); aslam.jarwar@hufs.ac.kr (M.A.J.)

**Keywords:** Internet of Things (IoT), Web of Objects (WoO), IoT analytics, microservices, semantic ontology

## Abstract

In the era of digital transformation, the Internet of Things (IoT) is emerging with improved data collection methods, advanced data processing mechanisms, enhanced analytic techniques, and modern service platforms. However, one of the major challenges is to provide an integrated design that can provide analytic capability for heterogeneous types of data and support the IoT applications with modular and robust services in an environment where the requirements keep changing. An enhanced analytic functionality not only provides insights from IoT data, but also fosters productivity of processes. Developing an efficient and easily maintainable IoT analytic system is a challenging endeavor due to many reasons such as heterogeneous data sources, growing data volumes, and monolithic service development approaches. In this view, the article proposes a design methodology that presents analytic capabilities embedded in modular microservices to realize efficient and scalable services in order to support adaptive IoT applications. Algorithms for analytic procedures are developed to underpin the model. We implement the Web Objects to virtualize IoT resources. The semantic data modeling is used to promote interoperability across the heterogeneous systems. We demonstrate the use case scenario and validate the proposed design with a prototype implementation.

## 1. Introduction

IoT analytics is evolving with new technologies, tools, and data processing and analysis methods in order to enhance our environments with increased productivity and improved efficiency. In IoT, smart sensing and actuating objects are coupled with information and communication technologies. They are embedded in digital environments to produce myriads of data which need to be extracted, processed and analyzed efficiently to support IoT applications.

Data analytics has been explored in various domains including business, healthcare, energy management, infrastructure handling, safety, security, etc. However, IoT analytics pose several new challenges. A few of these include processing of streaming IoT data coming from diverse devices, managing different data formats, real-time annotation of sensor observations to identify their meaning and context, and most importantly, processing and utilizing such data with reusable and easily maintainable services rather than monolithic approaches. These limitations affect the overall efficiency, scalability, and reusability of the analytics solutions in IoT. To face these challenges robust design models and methods are needed which can efficiently provide useful insights from IoT data.

A modular design methodology is highly required to support predictive analytics in a constrained computing environment like IoT. To realize such scalable and reusable design we adopt the microservices approach where every part of the analytic process is embedded in a service. In contrast to monolithic approaches, proposed design considers each service to handle a specialized functionality which is scaled and managed independent of other services providing enhanced efficiency and more resistant to failures in the IoT environment.

Several use cases show the significance of predictive analytics for various applications. These include, for example, predicting energy consumption patterns to manage electric infrastructure in a smart city scenario [1] or detecting activities from sensor observations in healthcare domain to provide services such as preventing fall in stroke patients [2]. Likewise, a use case in a smart home environment supports elderly people by predicting their behavior [3]. Data analytics is not only limited to sensor observations it also includes many soft aspects such as the analysis of social media data. In such cases, social data is obtained through crowdsourcing to detect urban emergency events [4].

In IoT environment, machine learning (ML)-based data-driven approaches are used to provide analytic capability for sensor data. Machine learning has become an important part of almost every system which needs the automation of its processes or identification of hidden patterns from the data. IoT systems are no exception where a very large amount of data is generated day by day from numerous sensors, which has spawned a demand for processing and analytics. Several works in the literature [5,6,7] and numerous developed systems [8,9] witness the power of ML techniques to provide predictive analytics solutions. Multiple types of sensor data stored in the repositories are extracted and refined to train ML models. ML solutions are proactive in the sense that they predict the events that are going to happen in the future based on training from historical data.

Even with the advent of several ML-based analytic solutions, efficient data analytic designs for IoT are still limited which can effectively predict and analyze events on heterogeneous data. Particularly the scalable models are rare that can support intelligent and modular analytic services based on ML techniques in IoT. These limitations motivate us to design and develop a system which can provide IoT services in an efficient and scalable manner.

The proposed design in this paper provides an enhanced analytic approach where data processing mechanisms based on machine learning and semantic web technologies have been considered. The two aspects distinguish our design in this landscape. The first one is the implementation of a model with a suit of microservices, where each microservice encodes a unique analytic functionality and shows interface through an API. These microservices can be modified and replaced independently to support robust and stable IoT applications with enhanced scalability. It has been realized that deployed analytics microservices are easier to compose due to a containerized environment. The second aspect is the semantic processing and annotation of sensor data which helps in the knowledge-based understanding of IoT data and supports analytic functions with inferencing on the annotated data in contrast to the raw data which lacks semantics.

Apart from providing the analytic capabilities, an IoT system should have a well-defined data representation mechanisms. In this regard, numerous approaches of innovative data handling solutions have been proposed and developed in the last few years. One of these approaches was given by European FP7 project iCore [10,11] which proposed a cognitive framework for IoT applications [12,13]. The iCore project discussed the notion of the virtual object (VO) [14] to represent ICT and non-ICT objects. However one of the limitations was traditional monolithic services to handle virtual objects. On the other hand, Web of Objects (WoO) framework [15] extended the VO concept to represent sensors, devices, information, and processes. It enabled semantic representation of Real World Objects (RWOs) in VO to promote extensibility and interoperability. WoO provides necessary guidelines for the development of virtual objects, their composition, and deployment to achieve IoT service functions in a web environment. In our proposed design we use WoO concepts to develop analytic features built on microservices.

Moreover, to provide a scalable design model which supports predictive analytics for adaptive IoT applications, this article focuses on the following key questions. How can modular analytic processes be developed using microservices? How can the streaming data be prepared, processed and analyzed efficiently in an IoT platform? How can the microservices be orchestrated with Virtual Objects (VOs) and Composite Virtual Objects (CVOs) to perform analytic functions? How can VOs semantically represent stream data? How can the analytic results be consumed by different IoT applications with standard interfaces? To summarize, this article provides the following key contributions:

- We present a design methodology based on microservices to support predictive analytics for IoT applications. We identify the major features of the predictive analytics and then, develop and deploy them into microservices. In the outcome, each autonomous microservice handles an analytic functionality where it is composed and orchestrated with other microservices in the containerized environment to achieve service objectives.
- The proposed design combines both the data-driven and knowledge-based approaches to process and analyze IoT data. In this view, IoT data processing mechanisms based on machine learning and semantic web technologies have been considered.
- We demonstrate a way to combine semantic technologies in machine learning pipeline with respect to feature creation from semantically linked annotated data.
- We have designed an ontology for the semantic representation of streaming data. Semantic web technologies are used to represent multi-source IoT data. We also present virtual objects and composite virtual objects to create virtual counterparts for physical devices in our implementation.
- We demonstrate a use case scenario and a prototype implementation where the components have been developed to realize the feasibility of the design.

The remainder of this article is organized as follows: Section 2 presents the related work with respect to analytics processes and microservices design for IoT application development. Section 3 presents the proposed design methodology of microservices to support predictive analytics for IoT applications. In Section 4, a use case scenario in an IoT environment is explained. Section 4 also includes details on the implementation prototype and performance evaluation measures. Finally, we conclude this paper in Section 5.

## 2. Background and Related Work

Literature renders several examples of analytic solutions for the IoT environment. Most of the approaches are based on ML techniques, these are further discussed in Section 2.1. On the other hand to enable modern IoT applications scalable service components such as microservices have also been realized, where the details are provided in Section 2.2. In this regard, this section presents the related work with respect to IoT Analytics and microservices.

### 2.1. IoT Analytics

In recent years, increased advances in sensor devices, communication technologies, and cloud computing have enabled IoT systems to collect data from diverse sources at an unprecedented scale. These large volumes of data have created tremendous opportunities to provide smart service features from an analytics perspective in IoT systems. Analytic features have become an important part for the IoT platforms to serve in diverse domains from energy management to infrastructure management, from identifying problems in the community to individual’s health tracking. Several use cases have been discussed in the literature to deliver IoT analytic services.

WANDA [16] is one of the IoT analytics systems that provides an end-to-end remote patient health monitoring and data analytics solution. The system collects sensor data based on a smartphone gateway and provides the data storage mechanisms. For the diagnosis of a patient’s health records, it uses an analytics engine to predict future events on the basis of physiological observations. Also, to support ambient assisted living, IoT analytics is explored in [3], where a framework integrates contextual information to determine the wellness of an elderly. In this work, authors have developed a behavior detection procedure based on sensor data related to daily activities. The system predicts the wellness of an elderly by analyzing the daily usage of appliances in the home. Another analytics system [17] in the healthcare domain provides a solution based on VIRTUS IoT middleware. The system aims to monitor the patient’s body movement and classify daily activities. 

An automated Internet of Things enabled model for on-shelf availability [18] uses sensors and analytics to analyze information from diverse sources in order to identify out of stock products on shelves and notifies the store contacts. This model detects and predicts trends related to the out of stock conditions. 

An edge intelligence framework to build IoT applications has been described by [19], where authors demonstrate the stream processing at the edge devices to support timely and reliable data analytics functionality. The authors focused on comparing performance issues between cloud and edge using an activity recognition module. On the other hand, an IoT analytics framework design consideration based on fog computing concept has been presented by [20]. The framework supports transitions of data between the edge and the cloud. However, the authors did not elaborate on the detailed mechanisms and they did not consider approaches to deal with heterogeneous data. Further works include some examples from industry such as in [21] authors have proposed a knowledge-based analytics and clustering framework for BI applications and authors in [22] have proposed a knowledge reengineering framework on IoT analytics for BI services. 

Although, several IoT solutions discussed above, offer analytics capabilities in order to deliver intelligent features at different scales. Most of them are modeled on highly coupled monolithic architectures and doesn’t seem to scale well. Still, many improvements are required in the form of modularity, reusability, and interoperability to achieve full benefits of these systems. 

Besides, microservices have become a design choice for improving IoT systems with modular and reusable analytic functionalities. Analytic features packaged into autonomous microservices can enhance the modularity and reusability of the system. In the following section, we define some of the IoT systems that utilize microservices concept in an IoT environment at different levels of their architectures.

### 2.2. Microservices

Most of the systems in the IoT domain have been developed with monolithic approaches in recent years. However, a new way of application development has been witnessed with the microservices pattern. The Microservice architecture has become a popular approach for the development of flexible distributed software applications [23]. In microservices based design small services are developed completely independent of each other with lightweight communication mechanisms to achieve business requirements [24]. Martine defines his opinion of microservices as: “Gather together those things that change for the same reason and separate those things that change for different reasons” [25]. This view suggests that microservices are services that are targeted on a single responsivity principle that means, doing one thing well at a time. Also, the microservices are autonomous units that can be deployed and changed independently from one another without affecting their consumers for any change [23].

Several studies show significant benefits achieved while using microservices. They have been incorporated in several IoT projects such as in [26] authors demonstrate the use of microservices for M2M applications to cope with the limitations of monolithic services design. Similarly, work in [27] elaborates the benefits achieved using microservices in large-scale application development for a smart city environment. Further, in the Almanac FP7 EU Project [28] microservices are used to foster service scalability for smart city applications in an IoT environment. The use of microservices has also been witnessed in the industry to develop large-scale applications. Such as Amazon, Netflix, and Pivotal [29] use microservices in their enterprise applications. 

Considering microservices for IoT projects is helpful in the sense to handle the complexity generated by a large number of IoT devices. With this approach, the system can be broken down into smaller parts to achieve lower coupling and to make a system more flexible for change. Development of the IoT system with microservices can provide several other benefits such as enhanced scalability with a decoupled pattern. Also, microservices make a system loosely coupled with plug and play behavior. The plug and play feature of microservices enables to add new functionality to the system or replace the existing failed service with a new one by pulling the plug from one microservice and attaching to the other [30]. Microservices can also be used to support the IoT system with their inherent problem of heterogeneity due to different devices and protocols. With the semantic web technologies, microservices can enable interoperable communication of information [31,32].

## 3. Proposed Design Methodology

The data analytics has shown very powerful applications with the innovation of new methods and techniques. To get insights from the data or to detect events of interest intelligent analytic approaches have been developed. However, to incorporate analytic features into an IoT framework requires a flexible and scalable service structure instead of traditional monolithic service development approaches which hinder the efficient IoT service implementation. To overcome this limitation and achieve a modular and scalable design we propose a design based on WoO reference architecture that realizes microservices with analytical features. We, further present the details of the design in the following sections. Section 3.1 presents reference architecture of the Web of Objects. Section 3.2 discusses different aspects of the proposed design.

### 3.1. Web of Objects (WoO) Reference Architecture for IoT Services

IoT is heading towards a vision to connect every object to the internet. However one of the major challenges is to deal with the heterogeneous objects which are packed in isolated technological and information silos followed by diverse communication protocols, different interfaces, and dissimilar data formats. There is the requirement of seamless interoperability across IoT systems to foster intelligent services. Several initiatives have been proposed in the past to achieve this requirement. Among them, iCore [10,13] provided the notion of VO to deliver digital representation of ICT and Non-ICT objects. The WoO reference framework extended the concept of VO to support IoT services over digital representations of diverse physical objects in the World Wide Web environment [33,34,35]. To provide interoperability in an IoT environment, WoO platform uses semantic web technologies and enables VO and CVO as web objects to support the IoT services [36,37]. The microservices in WoO design play an important role to provide modular service concept based on SOA principles. The multilayered WoO reference architecture is defined in [15] to support the development of IoT applications.

### 3.2. Details on the Proposed Design Methodology

The development of IoT and data-driven technologies is growing rapidly and influencing all technological areas with opportunities for industries and individuals. In big data analytics, researchers have contributed several works of predictive analytics to get insights from the data. However, dealing with data coming from resources constrained IoT devices and performing predictive analytics on such data in an efficient and scalable manner is still a prominent research area. In this paper, we have proposed a design based on the concept of lightweight microservices to moderately support analytic functions over the data coming from IoT devices. 

The proposed design is considered to satisfy the requirements of data analytics for sensor data as well as other types of information such as user profile history and social media events. The design incorporates the consideration to deal with efficient data processing for IoT applications. It supports the IoT applications to get insights from the latest incoming observations through sensors and correlate current data with the past stored history data to understand hidden patterns. One such example is to predict a user’s future behavior towards an event or a situation based on the historical records of his behavior. History data is important to analyze and generate responses to new situations, therefore it needs to be analyzed in advance to help services adapt to future requirements.

**Figure 1:**
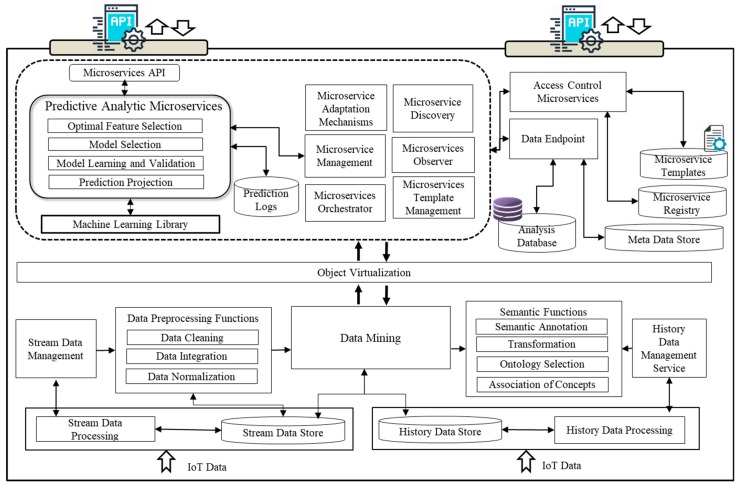
Design of microservices to support predictive analytics.

Figure 1 provides an overall view of the proposed model for IoT analytics with diverse components. The data collection items are distributed into two categories. The first category incorporates the sensor sources including all the input from sensing devices attached to the gateway. The second category of data sources contains user-centric information, such as a user’s profile, preferences, history, and social media representation. The personal information is acquired with the user consent. 

#### 3.2.1. Data Preprocessing and Transformation

Data preprocessing and enrichment layer provides two types of functionalities. The first one is the IoT stream data collection and preprocessing to prepare it for analytic procedures. The second part of the layer is the enrichment of data with the semantic ontologies to make it semantically interoperable and hence make it available for different IoT applications. In the first part of the layer, the Stream Data Processing Service receives data from IoT gateway using MQTT interface and this data is pushed towards stream data store. We have used Apache Kafka to process the IoT data in our design. Apache Kafka has been chosen because of the features and the integration support it provides with other technologies. Moreover, the stream data management service has been developed to perform preprocessing functions on the data which involves data cleaning, integration and normalization functions. In the data cleaning process, the missing values are filled with data imputation mechanism, noisy data are smoothed, outlier and inconsistencies from the data are resolved. The data integration function integrates the data originated from multiple devices into a uniform format. Data Normalization involves the transformation and aggregation of the values to normalized ranges to provide consistent data. Data Mining Function processes the prepared sensor data with machine learning models. These models are embedded in microservices to perform analytics functions over the sensor data. In the second part of this layer is the enrichment of the data with semantic annotations. The data is semantically annotated so that it can be utilized for further inferencing at the VO and CVO levels. Semantic functions include semantic annotation, transformation to semantic formats, selecting the appropriate ontologies to map the sensor and history data and association of new concepts in the selected ontologies.

#### 3.2.2. Virtualization 

The virtual layer provides functionalities that enable the digital representation of heterogeneous devices and the generated data in the form of virtual objects. Two key concepts of virtualization are VOs and CVOs. Several functional components facilitate the virtualization of objects. These include *VO Configuration and controlling function* which provides necessary mechanisms to discover VO instances in the repository. It registers and maintains the entries for VOs in the registry and handles VO templates. The *CVO management function* handles a more complex composition of VOs and generates system level knowledge to optimize VO utilization for efficiently achieving service layer requirements. *Data management component* at the virtual object layer manages the data associated with the VOs and CVOs. This functional component provides CRUD operations over the data and interacts with semantic databases. Object management component maintains object instances and manages the existing objects and their associations. It coordinates with the registry database to keep track of objects in the systems and maintains their association with other objects and services. The CVO and VO semantic data stores are designed in the proposed model to expose interfaces for stored objects in RDF graphs. The metadata of different virtual objects is accessible from the metadata store. The metadata is required to process objects based on their unique characteristics. 

#### 3.2.3. Service Support Functions for Microservices

The service support functions are defined to handle general functionalities for microservices in the proposed design. Few of these functions have been defined in our previous works [32,33] and are extended in this work to support predictive analytic services. These functions are related to discovery, configuration and management, and handling microservices templates. The Discovery function provides search capability by mapping identifiers and the instances in the Microservices registry. On the other hand management function involves the selection of microservices in response to the service requests as well as the identification and reuse of existing analytic microservices. The function maintains the configuration settings for each microservice. The Microservices Template Management provides an interface to store and retrieve templates which constitute information about the specific functionality of each individual microservice, including its associations and sub-functions. Moreover, analytics microservices use service support functions to use data under controlled access with data endpoints. Registry and repository contain references to available microservices and their service templates respectively. 

Apart from the above service support functions, some additional functions include orchestration management, microservice adaptation, and observer modules. An orchestrator enables integration of multiple microservice operations by preserving the output of each service towards a combined result. Microservices Observer collects and observes the data about the failures in microservice operations and keep a check on remote calls and the sensitive parts which need to be watched to provide protection from failure. The adaptation mechanism handles the adaptation rules which affect the microservices based on environment. The concept behind adaptation is to execute new code defined in the rule which is based on the condition and type of microservice.

#### 3.2.4. Predictive Analytics in Microservices

**Figure 2:**
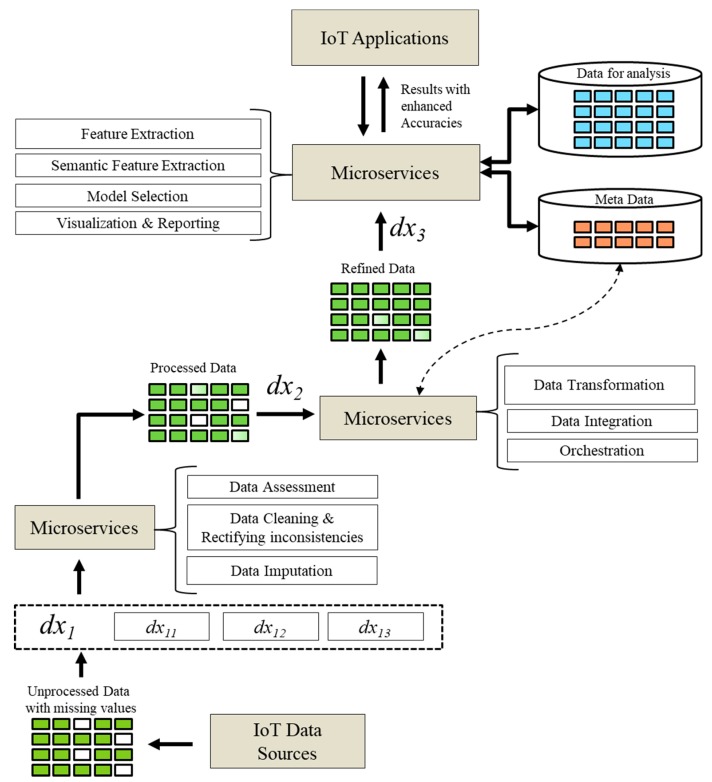
Procedures to achieve predictive analytics.

This section describes a general overview of the processes embedded in microservices to perform analytic functions. The details on these functions are elaborated in Section 3.2.5. The prediction results are evaluated based on the steps shown in Figure 2. Initially, the acquired data from sensors are unprocessed, containing missing and inconsistent observations which we represent here as the dx1 matrix. First, the proposed model improves the data to achieve better analytics based on the principle of the higher the quality of the data, the better the results produced by analytic procedures. The unprocessed IoT data is enhanced with preprocessing method (for which details are provided in Section 3.2.5). The dx1 is provided to the data preprocessing microservices, which assess the data for any lost or inconsistent values. The microservices clean the dirty data and rectify the missing observations and inconsistencies. In case of missing or lost data, imputation is performed to recover the values. The data preprocessing microservices forward the processed data (expressed as dx2) to higher level microservices. The processed data is partially ready for performing analysis, however, to achieve higher accuracies, microservices perform normalization and transformation functions. They also integrate heterogeneous sourced data and enrich them with metadata. The orchestration is performed by microservices if many VOs are involved in the process. Microservices generate a refined data matrix expressed as dx3. The refined data matrix is provided to the analytic processing microservices which perform machine learning to deliver the results.

The microservices are containerized services where each one of them is independently providing functionality in an isolated manner. These functionalities combined through a pipeline of several microservices aim to meet overall service objectives. The IoT analytics is supported with the machine learning development since machine learning mechanisms have become a prominent part of the intelligent IoT applications. The main tasks of microservices include preprocessing, feature extraction, model selection and validation, semantic feature extraction and selection, and visualization. In our system, microservices are exposed using the microservices API. Microservices extract the features from the dataset to achieve better results with higher accuracies. These microservices encode algorithm for feature extraction as discussed in Section 3.2.5. Other microservices acquire features from the semantic data coming from the CVOs and VOs. In order to achieve accurate results, features are further enhanced with statistical methods. Once the features of interest are selected by the microservices, the second step is related to the use of these features in the learning process. The learning process involves the model selection, where microservices choose the appropriate learning model that is trained over the data and provides a low error rate with testing data. The learning microservices prepare the models with the help of machine learning algorithms where the most suitable algorithm is selected or a combination of several algorithms is adopted. The Projection Microservices test the selected learning model with the extracted features based on the test dataset. They recursively perform analysis on the data to achieve the highest prediction accuracies. The predicted analytic results generated from these microservices are used by the Visualization Microservices that generate more specific views to get insights from the data.

#### 3.2.5. Analytic Processes Pipeline

##### Data Set

The data used here is PAMAP2 Physical Activity Monitoring dataset [38], which consists of 18 diverse physical activities such as walking, cycling, etc. The dataset includes observation acquired from nine individuals wearing sensors like inertial measurement units (IMUs) and heart rate monitors. The data set consists of 54 attributes with activity ids and different sensor measurements. The reason for choosing this data set is because of the sensor observations which are similar to the IoT data in our use case scenario. The details of attributes for the dataset are given in the Table 1.

##### Data Preprocessing

**Figure 3:**
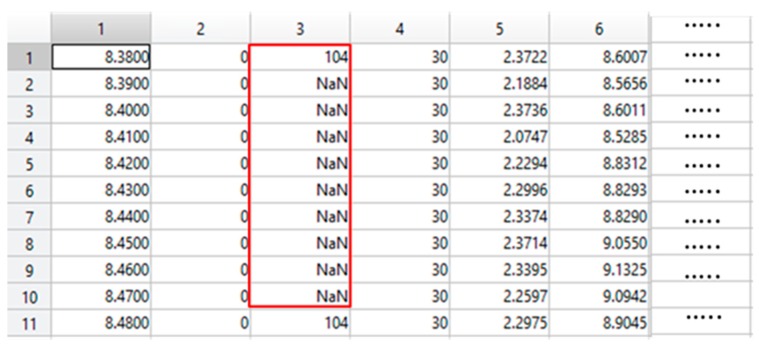
An example of window selection to impute missing values.

In the data pre-processing step we perform a check on missing values and it is observed that a large number of heart rate and skin temperature values are missing. First, we tag these values as NAN as shown in the snapshot of the dataset in Figure 3. The missing data are imputed using a time window based approach [39]. However, we impute data based on the last value that is available in the specific window. This technique is found better because values are not frequently changed. In the above dataset, we use the sensor measurements from the IMUs such as IMU values for hand and chest.

##### Feature Extraction

**Figure 4:**
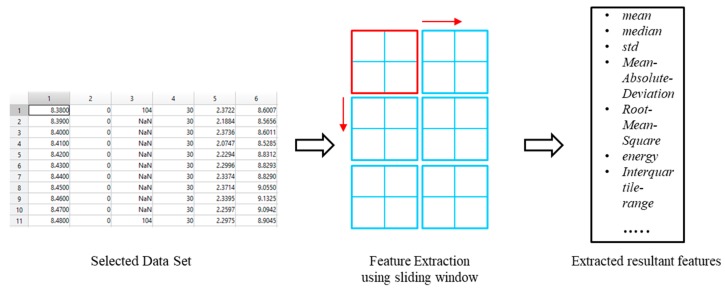
Feature extraction process.

Feature extraction has been performed by computing the features from sensor data set (PAMAP2). Features extracted from the dataset are based on IMU and heart rate and skin temperature observations. The approach of feature extraction has been carried out similar to the other studies on activity recognition [40,41]. The feature extraction is performed by defining windows on the dataset attributes as shown in Figure 4. The size of window differs from one scenario to another, and in this case, we have used a fixed window size of 15 samples and an overlapping of 30% among windows which provided us good results. The feature extracted with respect to IMU include mean, median, standard deviation, Mean Absolute Deviation, Root Mean Square, energy and interquartile range. Some of the feature extracted from heart rate data consist of the mean, scale of change and trend.

##### Creating and Selecting New Features from VO Data

The linked semantic data provides a background knowledge which improves the analytic process. Enhancing the dataset by taking features from semantic web data improves the analytics results in many cases. It also supports to maintain the knowledge about particular domain externally. Here we extract the features from the semantically linked VO data. Extracting features on linked data has been explored in other studies as well. Such as FeGeLOD [42,43] creates data mining features from the linked open data to augment data for the mining process. Another study [44] provides a system that integrates data mining on the semantic web by enabling the user to retrieve features from linked open data that can be used by machine learning methods.

Generally, machine learning techniques compute analytic result from the data that are represented as a propositional feature vector in which each instance is presented as a vector of features V = {f1, f2, f3, …, fn}. On the other hand, linked semantic data are described as graphs where data in the form of entities and a set of relationships are maintained which comply with a domain ontology.

**Figure 5:**
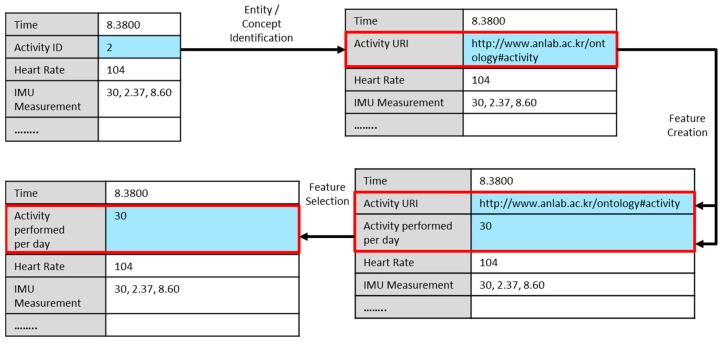
Example of feature selection from linked semantic data.

To extract useful features from semantic web data which further can be used by statistical machine learning techniques for analytics, a three-step process is followed similar to the one used by [43], the difference in our case is that data is semantically represented in virtual objects. In this approach, the first step is to identify the data instance and then identify the corresponding entity in the semantic data graph associated with that data instance. The second step is to generate new features from the identified entity and third is to filter and select only the relevant features from a feature set. These steps are briefly discussed in the following section and the example instances as illustrated in Figure 5.

Recognizing the entities—The concepts in the linked data are identified as URIs. For example, an activity concept in the semantic data set is represented as http://www.anlab.ac.kr/ontology/activity. To acquire meaningful feature from linked data we need to identify the URI that is related to one of the entities in our dataset which is “activity” entity in this case. The example shown in Figure 5 depicts that the activity id is resolved to URI of the “activity” entity, which is selected as a new feature. 

Creating new Features—After identifying the URI of a particular entity we use it to generate features. The feature generation process reads each data property of the concept and generates a feature. Data properties are defined as values like the name of the activity or the frequency of activity (number of times an activity has been performed). As illustrated in Figure 5, the feature generation process performs a query on the RDF graph to access the property of “activity performed per day” and a new feature is extracted. Several other types of features can also be extracted based on the type of entity. RDF enables an entity to be defined of several types. Such as activity entity can also be the type like action or task etc. There are several other ways to create features from the semantic web data which are being investigated in many studies [45]. 

Selecting features—Creating features from an RDF graph can yield a huge number of features depending on the criteria of the feature generation process. For example, in the above example, we used data properties to generate new features. In this case, a large number of data properties will create that much large number of features. However, all generated features are not useful for the analytic process therefore important features need to be extracted. Feature extraction problem has been investigated in the literature extensively and there are many solutions [46]. For the feature extracted from the RDF data, we have used a simple heuristic, where we discard the features that have the highest number of missing values. In this case, we have defined a threshold of T = 90%. That means from the new features we select only those which have not more than 90% of missing values. 

##### Semantic Annotation of Data

**Figure 6:**
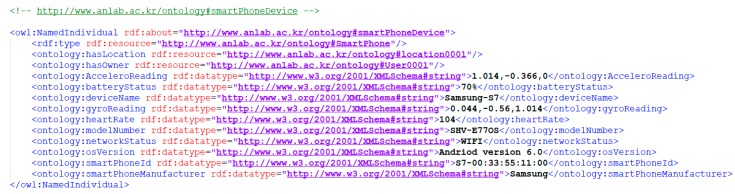
Semantic representation of smart phone device VO.

**Figure 7:**
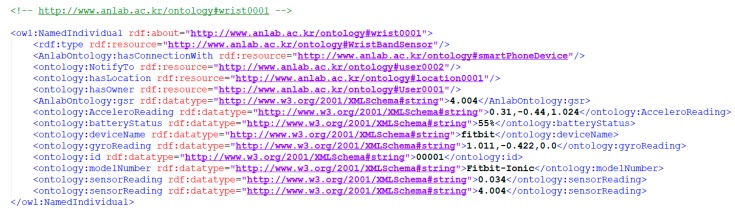
Semantic representation of wrist wearable device VO.

Collection of sensor data is crucial in many scenarios, such as assisting a medical diagnosis for patients or remotely detecting an elderly in an emergency situation like fall detection. Semantically annotating such sensor data with already well-defined knowledge in ontologies enables efficient analysis and integration. In the previous section, we defined how the data from sensors can lead to recognizing some activity with a data-driven approach. Similarly, the knowledge-based approach enables the inferencing on semantic data through inference engines to identify the hidden facts. We show how the sensor readings from smartphone device (shown in Figure 6) and wrist wearable device (shown in Figure 7) are semantically annotated. We also mention that we consider the data from smartphone sensors and wearable band sensors similar to the IMU and heart rate data in the PAMAP2 dataset, which are represented in the semantically annotated formats in VOs based on the defined ontology. The following descriptions describe OWL representations for the smartphone and wearable sensor devices where the devices attributes and their sensors observations are annotated with the ontology.

#### 3.2.6. Analytic Processes Flow

**Figure 8:**
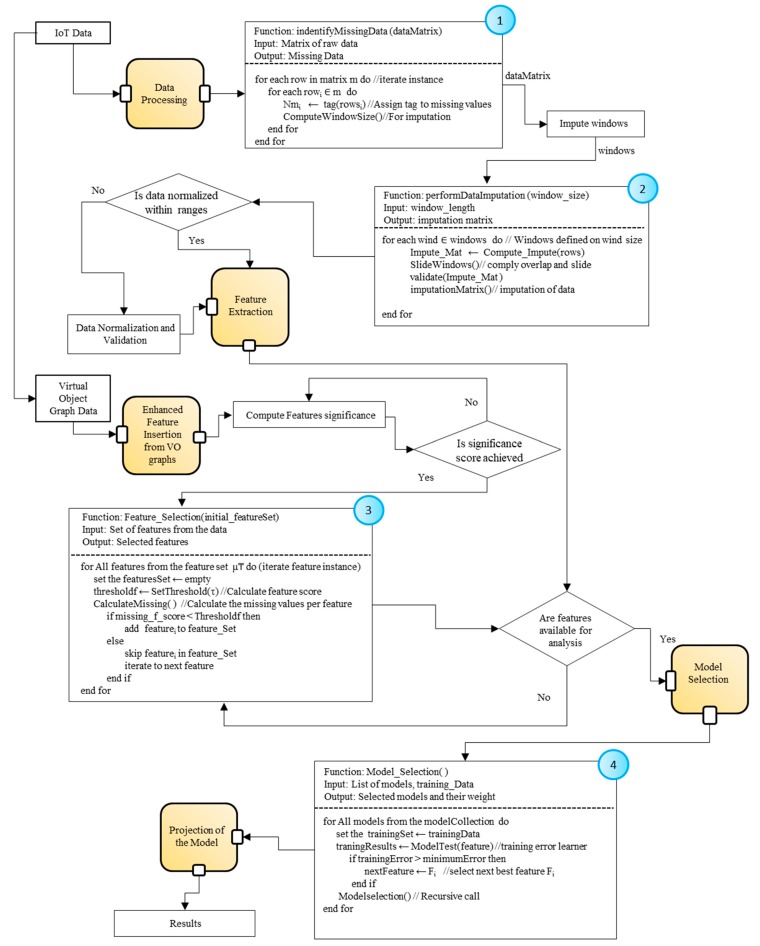
Analytic processes flow diagram.

The steps shown in Figure 8 presents the tasks performed during the analytic procedures. A brief heuristic for each task has been shown separately in the flowchart diagram. The first task is the extraction of raw data from the stream data store. The data preprocessing mechanisms are embedded in the system which identifies the missing data from the raw data matrix provided as input. The identifyMissingData (rawDataMatrix) function iterates the rows and tags those that contain missing values. The perform DataImputation() function takes an input “window_size” of missing tuples. This function executes the procedure based on a time-based window to impute missing value in the data, applicable to our selected PAMAP2 dataset. As discussed earlier we impute data based on the last value that is available in the specific window, because the values are not frequently changed. The function further validates a matrix with the imputed values. After the successful imputation of data, if required a normalization procedure is performed. The normalization is a processing technique or a scaling technique used to formulate the data in the same ranges in order to reduce the biased data values. Once data are prepared using preprocessing mechanisms, the next step is of extracting features of interest from that data. The feature extraction process analyzes features of interests where feature extraction is performed by defining windows on the dataset attributes as discussed in earlier Section 3.2.5. Moreover, we have enhanced the feature set by inserting new feature from RDF based VO graphs. In Figure 8, the heuristic of step 3 shows the process of feature extraction from RDF data, we discard the features that have the highest number of missing values. In this case, we have defined a threshold of T = 90%. That means from the new features we select only those which have not more than 90% of missing values. Further details are rendered in Section 3.2.5. Besides, the model selection microservice selects the model and tunes and validate the model for a prediction on the data. It implements the Model_Selection() function which takes the initial model instances and the training data. The function trains the selected models whereas the minimum error rate is considered the criteria for each selection. The implementation of analytic microservices serve as autonomous components which encapsulate automated learning methods and these are orchestrated in the composition based on the IoT application requirement.

#### 3.2.7. Microservices Composition

The analytic process needs a composition of multiple components which requires identifying microservices that can be composed in an analytic process and generate a composition graph. The Algorithm 1 (with the details on notations presented in Table 2) provides a pseudocode of the program for microservices composition. Two inputs are provided to the algorithm, the first one is the collection of microservices and another one is the entries of microservices in the registry. In the algorithm, a list of microservices is extracted and loaded in the model represented as ⅀Mμ and a list of virtual objects is loaded in the object model ⅀Mo. The lines 3 and 4 perform a query on the prepared model and iterations from line 5–11 identify the matching microservices as given in the pseudocode. The loop iterates on each of the matched microservices from line 12–19 and checks the available microservices that can be used in the composition. The rank of microservices is evaluated based on already defined criteria. In lines 20 to 27, the ranked microservices are composed in a workflow based on the inputs and output of microservices. The queue is formed and workflow is stored in the composition graph (Wf_Compositions) to be reusable for services.


**Algorithm 1: Composition of Microservices**
**Require**: A set of microservices (⅀ф) and the pointer to entries in the registry (ℝ_e_)**Output:** Microservices Composition Graph (**ℂ**) 1: ⅀Mμ ← Load list Microservices extracted from the registry in the model 2: ⅀Mo ← Load list of Objects (CVOs, VOs) in the model 3: μ₸ ← *performQueryOnServiceModel* (⅀Mμ, qr) 4: Ώ ← *performQueryOnObjModel* (⅀Mo, qr’) 5: **for All** $x_{i}\in \mu ₸$
**do** 6:   **for All**
λi∈x
**do**
 7:     **if**
λi=any
*ins* Ώ in Ώ **then** 8:       add λi to ℿ_matched_ 9:       persist ℿ_matched_ into selected composition graph10:     **end if**11: **end for**12: **for All**
***μ***
∈ℿ_matched_
**do**13:   **if**
$\mu_{i}\in AvbMat$//Check the microservice availability matric14:     **if**
$\mu_{i}=any\;\mu_{i}$ in ℿ_matched_
**then**15:        Ɵ ← *Evaluate_Ranking* (Rank assignment for microservice)16:        add $\mu_{i}$
**to** ℿ_MRanked_17:        store ℿ_MRanked_18:   **end if**19: **end for**20: **for All**
σj∈ ℿ_MRanked_
**do**21:   **if**
σj(Rth)≥ε
**then**22:     include σj to Wf 23:     Ƥ ← Wf//Add Workflow to Priority Queue24:     *Validate_Queue* (Ƥ)25:     Store Wf to the List of Wf_*Compositions*26:   **end if**27: **end for**

#### 3.2.8. Microservices Design with Web Objects

**Figure 9:**
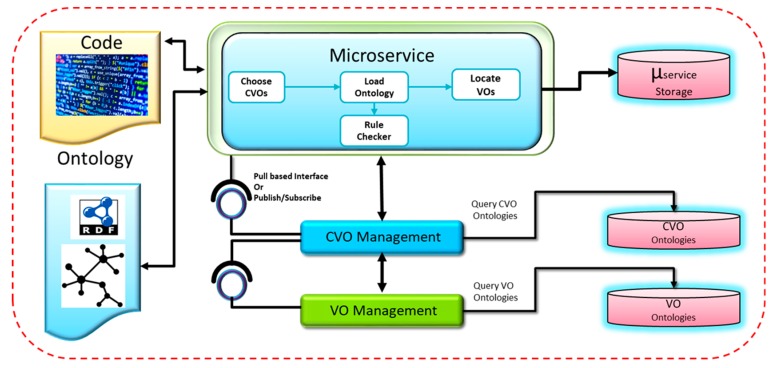
Design of microservice with Web Objects.

To analyze IoT data in WoO framework [47], microservices have been designed with specific data processing functionality. The instantiation of microservices involves selecting the CVOs from the available CVOs in the repository that provide a particular mashup of VOs. In case no CVO is available to handle the request a new CVO is created using an initial CVO template. The microservice also locates the VOs that represent sensors and other information by interacting with the CVO ontology. The rules that can be applied to VOs are checked and enforced on the VO data. The adaptation procedure in the CVO selection process is applied in which microservices dynamically select the CVOs that are most suitable depending on the service request and also the current context. To handle VOs and CVOs, management components provide RESTful and publish-subscribe interfaces. The necessary components for the instantiation of microservices are illustrated in Figure 9.

## 4. Discussion on the Use Case and Prototype Implementation

### 4.1. Use Case Scenario Details

**Figure 10:**
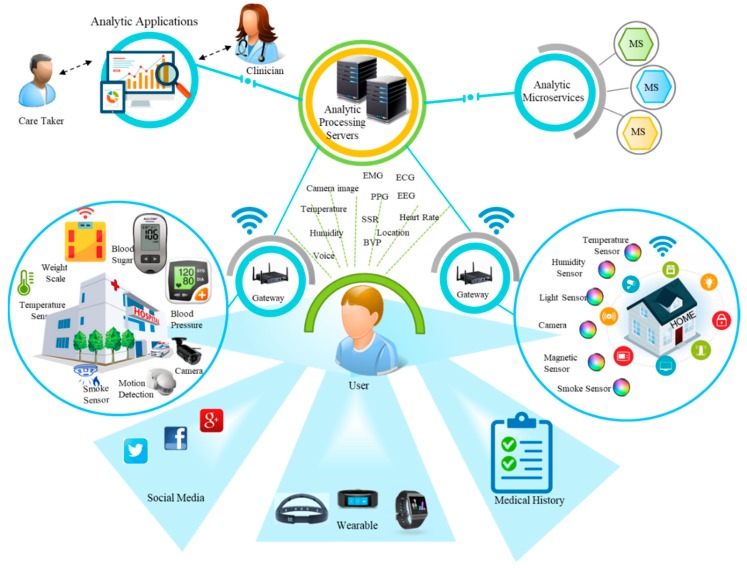
Smart health environment use case scenario.

This section presents a use case scenario about monitoring health conditions in an IoT environment. Assessing people’s health based on the data extracted from smart sensors can provide key indicators which are helpful to make timely decisions. The abrupt change in one’s health condition can have sometimes severe consequences. Identification of physiological conditions from diverse data sources such as data from IoT sensors and examining this data with analytic services can provide opportunities to predict health conditions and deliver innovative services that can support one’s health and avoid serious situations. We consider services supported with predictive analytics using microservices design in a smart health provisioning use case. The use case scenario is depicted in Figure 10 to represent high-level elements including data sources, processing system and consumer applications with smart home and health facility IoT infrastructure. The sensing environment includes physiological sensors connected with gateways. Other data sources include user social network services (SNS) data accessible using APIs and the user medical history to identify his health. The data from the user-wearable devices and home and health facility sensors are pushed towards gateways using RESTful and MQTT interfaces. The microservices running on analytics processing servers analyze the user data with machine learning mechanisms to provide insights into health conditions. Each microservice encodes a unique functionality such as the personalization microservice handles a person’s profile and history parameters. This microservice analyzes a user current context and maps it with the previous history to identify the choices for the user and provides such data to other services using an API. Another microservice recommends a user with different suggestion options based on his health condition such as in case of depressed mood recommending favorite movie and music, or suggesting to connect with a close friend or family member. The notification microservice notifies health status in case of the serious emergency situation based on triggers detected from user data. Social data analysis microservices analyze the social media data of the user with the API support and analyze the sentiments from the user data. The positive or negative sentiments are treated according to the service conditions set in the microservice design.

The above microservices make use of specific VOs and CVOs which represent the data received from the devices and provide conditions to produce actionable information and enable the analysis of the gathered data, either it is coming from sensors or collected from other sources.

**Figure 11:**
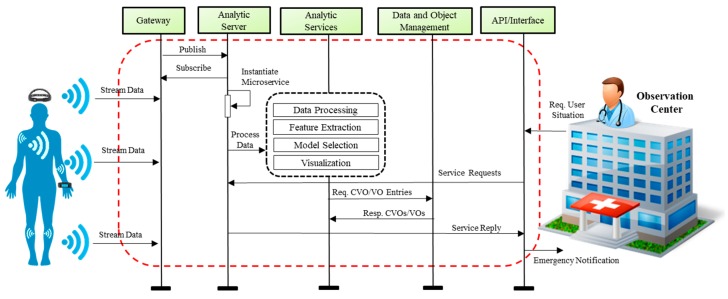
The flow of events for data gathering and analytic service provisioning.

In the proposed use case the core part is to provide analytic services to the end user, especially to the one who is interested to observe a patient’s current health condition and predict his future situation. The flow of operation in the proposed use case can be seen in Figure 11. The devices associated with user provide an interface to extract data using the gateway where this data is made available through publish/subscribe mechanisms. The analytics server subscribes the stream data and initiates the analytic process. The virtualization function in the use case is supported with VOs and CVOs which help to form the composition of multiple service features in analytics services. On the other end of the system users such as clinicians or caretakers from the observation centers can access the situation of particular users. The observer user can also request automatic emergency notification or alert services to effectively respond in case of emergency scenarios.

### 4.2. Prototype Implementation Details

**Figure 12:**
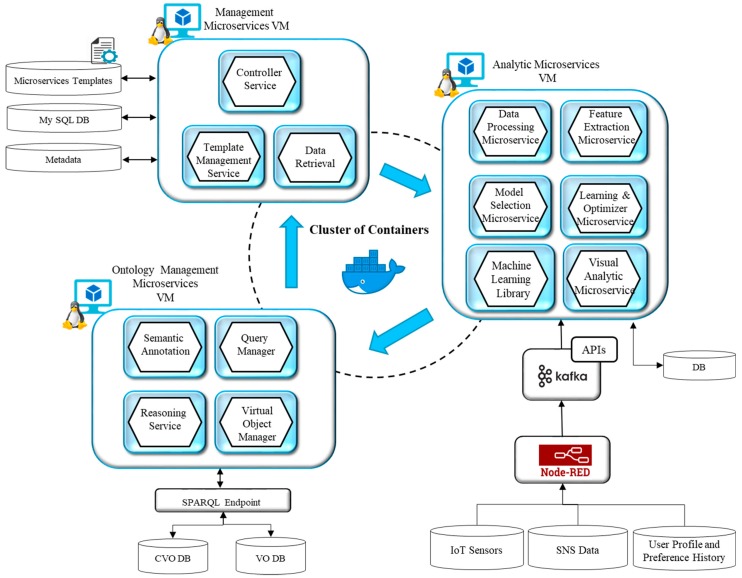
The prototype implementation system settings.

To realize the proposed design of microservices for predictive analytics in IoT, an implementation prototype has been established as shown in Figure 12. The design has been implemented and tested using available open source components. The scalability and reliability aspects have been considered in the implementation. The integration of machine learning components with microservices have also been well designed to provide analytical services.

Three virtual machines on Ubuntu system have been prepared with deployed docker images [48]. Each instance of the docker installed on the virtual machine hosts a microservice. The installed docker images together form a cluster of containers. 

For the orchestration of containerized services on docker, several solutions are available such as Kubernetes, Swarm and Mesos. However we have used Dockers own home-grown implementation for the container orchestration, that is the Docker Swarm. The reason for choosing Swarm is because of its lightweight solution for the container orchestration. The Docker setup is deployed on a machine with a configuration of Core i7 processor and 16 GB of RAM, running Linux OS. In Docker, Swarm Orchestrator deals with elements such as nodes and services. The node is the Docker engine’s instance that can either be in a manager or worker role. As a manager, it provides management, update, and termination of containers whereas a worker it provides hosting of the containers. The service defines what functions are to be executed on the workers.

The first installation of the virtual machine is dedicated to the analytic processes which include data processing microservice, feature extraction microservice, model selection microservice, learning and optimizer microservice, data visualization microservice and a machine learning library. The data processing microservice implements data preprocessing functions such as identifying missing and inconsistent data and processing it with data imputation and normalization methods. The feature selection microservice provides feature identification and selection functionality that can support the learning process with enhanced accuracies. The model selection microservice selects the most suitable model from the machine learning library and trains that model based on the model’s parameter settings. This microservice is supported with learning and optimization microservice which provides optimization function with pre-coded settings. The visual analytic microservice implements the visualization functionality for predictive analytic results acquired from the selected learning models. 

The second installation of virtual machine hosts microservices for service management functions. The microservices designed in this category include the Controller microservice, Template management microservice, and Data retrieval microservice. The controller microservice is at the core of management services which manages, monitors microservices and spots failures among microservices. If something is observed as wrong during execution such as abrupt failure of any service, it starts to replace the failed microservice with another replica and try to recover from failure. The template management microservice handles the template store and supports the instantiation of new objects from existing templates. The data retrieval microservice manages the data coming from different data sources and the associated metadata about devices and services in the metadata repository. It is also used to query data from the data sources and extract analytical results produced by analytic microservices. 

**Figure 13:**
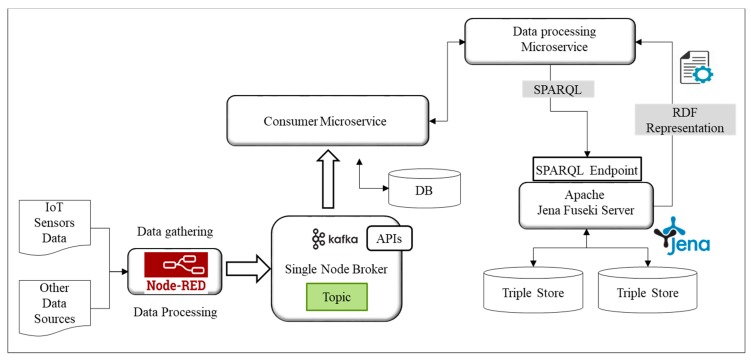
Data collection and semantic processing implementation diagram.

In IoT environment data comes from diverse sources with different formats. This data is extracted from external data sources using RESTful or Publish-Subscribe mechanisms like MQTT. Several data formats can be used to represent IoT data, however, most generally used formats include JSON and XML. In our proposed model we have chosen Node-RED [49] to collect data from different data sources. The Node-RED is an open source visual programming tool for wiring IoT devices, services, and APIs. It provides the capability to develop wrappers for dissimilar data sources and delivers APIs to connect the components with the code that is provided by the user. The data collected with Node-RED from IoT devices include sensor data which represents attributes like a user’s physiological state, SNS feed, user profile, preferences and response to the questionnaires using personal devices like a smartphone. RESTful API and MQTT are used to collect the data from these sources. Preprocessing is done at this level and the data is further pushed towards processing where Apache Kafka [50] has been used. It is an open source tool that provides message broker functionality. It enables publishing and subscription of messages to particular topics and maintains the data in these topics. It allows data producers to write to the topic using a producer API and the data consumers to read from it using a consumer API. In proposed implementation, the consumer microservice uses the Kafka consumer API to read from the sensor data topics. Moreover, in our case, we have used the data coming from IoT devices represented by the topics and used Kafka Connector API for building reusable consumers to connect topics to data processing microservice as shown in Figure 13. The reason for choosing Apache Kafka is due to the fact that it provides a scalable architecture with low latency and high throughput.

On the other hand, microservices use the semantic model of VOs and CVOs which have been developed using Protégé [51]. Apache Jena framework has been used to maintain VO and CVO representations. The Ontology management microservices VM deploys microservices that perform functions related to virtual objects. These include virtual object management, semantic annotation, query management, and reasoning services. Virtual object management microservice is an intelligent component that keeps track of VO and CVO instances and maintains their graphs in the respective repositories. The semantic annotation microservice annotates the sensor data with respect to the semantic concepts from the developed ontology. It transforms the sensor data with RDF/OWL formats. The query manager microservice generates queries on VO and CVO graphs using the SPARQL query language and extracts and updates RDF triples on SPARQL endpoints. The SPARQL endpoints are implemented using Apache Jena Fuseki SPARQL Server [52]. Fuseki integrated with triple store component has been used to provide persistent storage layer. We have used Fuseki version 2 on Java 8 in our implementation set up. The reasoning microservice encapsulates the reasoning function to provide inference over existing CVO ontology using the rules already modeled. The Apache Jena inference API has been used to infer new relationships among concepts from the existing modeled concepts.

Apart from the semantic data, to persistent the sensor data, MySQL DB has been installed. Sensors that have been used in the prototype include wearable devices from Fitbit that keep track of user’s vital health parameters. These include a heart rate monitor, accelerometer, gyroscope, altimeter, vibration motor, GPS and ambient light sensors. Moreover, SNS data feed has been collected using Twitter APIs. The user profile and preference information are recorded with a profile management microservice which persists this information in the user database and updates it on the user demand.

Further, in order to incorporate the learning mechanisms, Scikit-learn [53] machine learning library has been used. It includes several classifications and regression models for the prediction on the data. The microservices are coded to use these models in order to provide predictions.

#### Semantic Ontology

**Figure 14:**
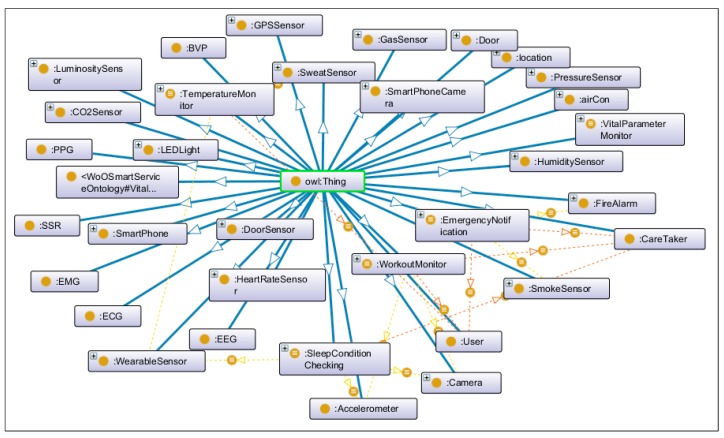
Semantic ontology for the proposed design.

In the developed system, CVOs and VOs are semantically interwoven using ontological relationships. These relationships are based on well-defined concepts in the proposed ontology as depicted in Figure 14. The semantic ontology allows incorporating rules and conditions on the data that dynamically trigger actions when thresholds cross defined criteria, such as emergency notification CVO composed in microservice triggers an event if the situation is recognized as an emergency based on the data coming from sensors. Several CVOs have been developed including Vital Parameter Monitoring CVO that monitors the vital parameters extracted from VOs such as blood volume pressure (BVP), electrocardiogram (ECG), electromyogram (EMG), galvanic skin response (GSR) and body temperature. The Emergency Notification CVO monitors the conditions on VOs to indicate any emergency situations. The Sleep Condition Checking CVO extracts parameters from the wearable and infrastructure attached VOs to analyze the data about a user’s sleep. The temperature Monitor CVO includes the identification of VOs data associated with the body temperature and the indoor temperatures. The Workout Monitor CVO analyzes user’s workout routine with the VO data coming from body wearables. Each CVO is a composite collection of many VOs, therefore a CVO provides several rules based on the data of VOs as each VO represent different device or information endpoints. 

**Figure 15:**
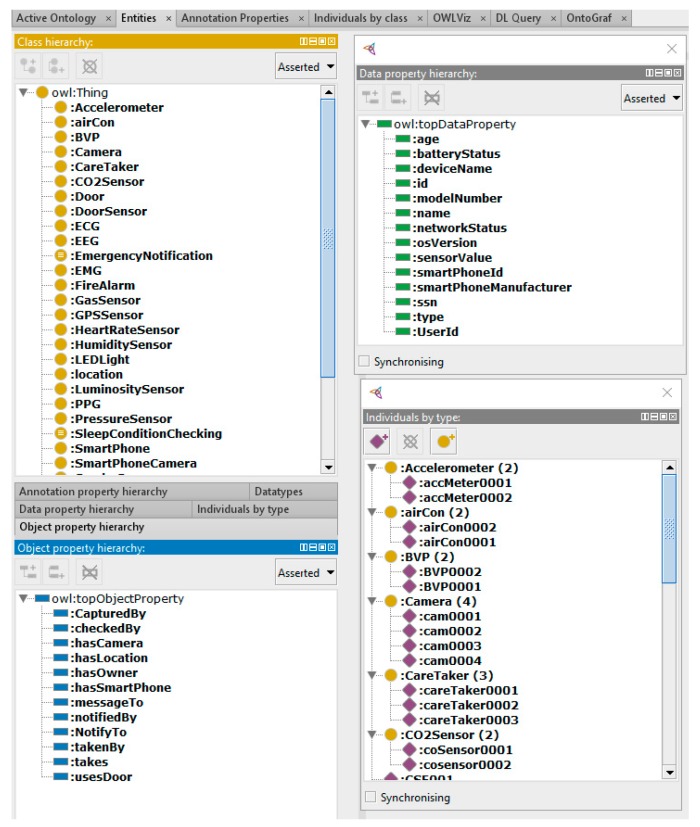
Views of developed ontology concepts in Protégé.

The development of CVO and VO semantic models have been performed using Protégé modeling tool. The views on the class hierarchy, object properties, and data properties are rendered in Figure 15. To support the operations on the object models, we used Apache Jena. To achieve CRUD operations on the defined models SPARQL has been used which supports actions on the semantic data, such a creating, modifying and deleting existing semantic RDF data. The link between semantic concepts is developed using the object properties. On the other hand, data properties incorporate the values specific to the type of each object in the defined RDF graph.

### 4.3. Results and Discussion

The main importance of performing following evaluation is to analyze the system’s analytic capability from a practical point of view on real-world data. We have used two healthcare domain datasets to train machine learning models in order to evaluate the prediction performance on the test data. The reason for evaluating different ML models is to realize that the models which have high accuracy will be evaluated and selected by microservices in the model selection process. The model selection microservice will perform a selection operation based on the prediction error of ML models on a dataset. The model with a low error rate and best accuracy will be selected by the microservices. Further, the performance of ML techniques will be directly related to the overall performance of analytic processing microservices that choose the model. Moreover, the evaluation of the system from service performance perspective has been rendered to demonstrate the execution and query processing capability of the system in order to perform well in an IoT environment.

#### 4.3.1. Analytic Results with Datasets

##### Analysis with Cleveland Heart Disease Dataset

**Figure 16:**
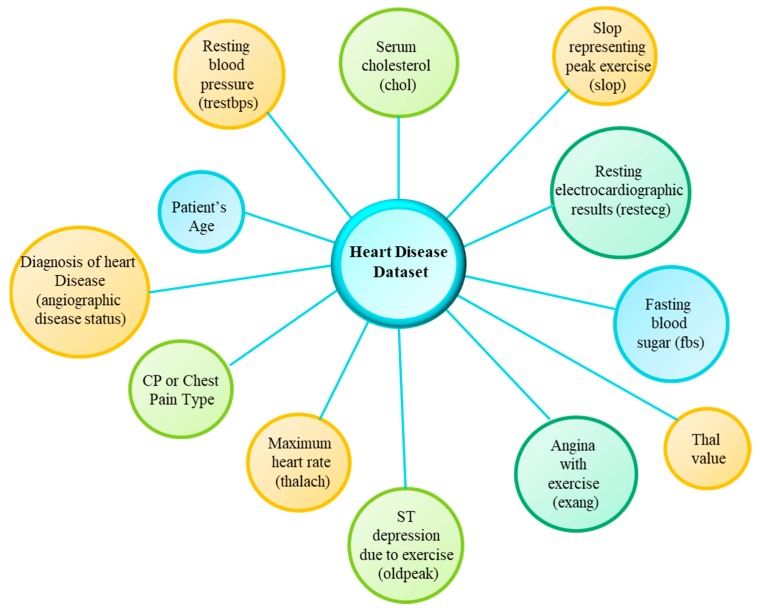
Heart disease dataset attributes.

A system which can provide monitoring and analytics capabilities for the patients with high risk of heart failure is highly useful. In this section, we discuss the analysis performed on the heart disease dataset. We have used Cleveland heart disease dataset [54] which was created by Detrano at the V.A. Medical Center (Long Beach, CA, USA) and the Cleveland Clinic Foundation (Cleveland, IL, USA). This dataset is based on more than three hundred samples. The data samples provide information about healthy and severe condition cases of heart disease. The dataset provides 76 types of attributes including the 14 highly useful attributes are shown in Figure 16. The range of values with the description of each attribute is described in Table 3. The dataset contains missing values which are handled with preprocessing in order to process the data before preparing the training and testing sets.

For heart disease dataset, feature extraction is performed similarly to the feature processing we performed earlier in Section 3.2.6. Based on the measurements taken in the dataset new feature have been extracted. The four new features include the mean, median, standard deviation and mean absolute deviation. 

**Figure 17:**
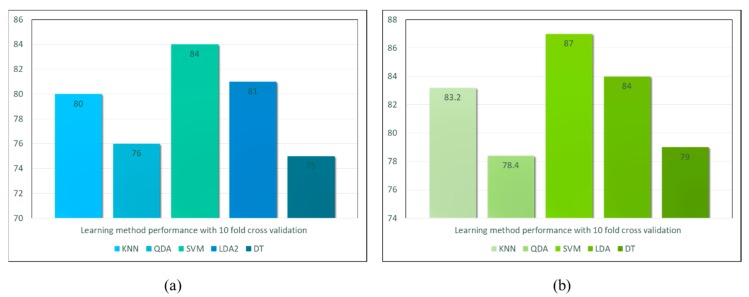
The performance results of different learning methods for Cleveland heart disease dataset (**a**) shows the accuracies with unprocessed data (**b**) shows the accuracies with processed data.

Moreover, based on the feature set, different machine learning models have been tested on the data, such as the support vector machine (SVM), the linear discriminant analysis (LDA), quadratic discriminant analysis, K-nearest neighbors (K-NN) and decision trees (DT). Each of the learning models is trained and tested on the data from the heart disease dataset, where different data has been selected for training and testing with a ratio of 80:20. The learning methods are evaluated based on 10 fold cross-validation. The results are illustrated in Figure 17. The accuracy of different models have been observed in two cases, first with unprocessed data (Figure 17a) and second with the enhanced data (Figure 17b). It is observed that compared to other models SVM outperforms them with 87% accuracy, whereas LDA provides a maximum of 84% and K-NN provides around 83% accuracy in the results.

##### Analysis with Diabetes Dataset

**Figure 18:**
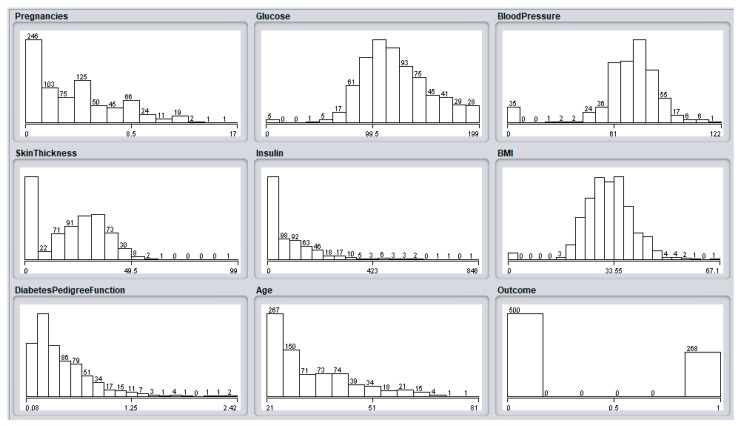
Data distribution for diabetes dataset attributes.

The diabetes data was collected from Pima Indians by the National Institute of Diabetes and Digestive and Kidney Diseases [55]. It constitutes more than 700 cases with eight attributes. The data is used to train the classifier to classify positive or negative diabetes patients. The list of eight prediction attributes and one class attribute is provided in Table 4. An initial data distribution for the diabetes dataset is shown in Figure 18.

Apart from the feature provided in the above table, we have extracted seven new statistical features on the measurements in the data set, these new features consist of mean, median, standard deviation, mean absolute deviation, root mean square, local maxima, and local minima.

**Figure 19:**
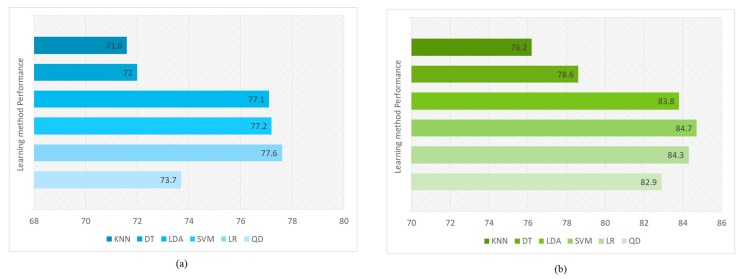
The accuracy of learning classifiers on diabetes dataset is shown in the graphs (**a**) Learning algorithm results before enhancing the data (**b**) Improved results after enhancing the data with data processing methods.

Furthermore, six classifiers have been used to classify the patient’s status with above 80% accuracies. The classifiers are trained and tested on the diabetes dataset and they are evaluated based on 10 fold cross-validation. These classifiers include SVM, LDA, Logistic Regression (LR), DTs, and K-NN. A maximum accuracy of 77% is achieved with different models on data before any enhancements to them as shown in Figure 19a. Additionally, after enhancing the data through the proposed system, a significant increase of accuracies was observed as shown in Figure 19b.

The evaluation of the algorithm performance has been done by identifying some statistical measures such as true positive (TP), false positive (FP), false negative (FN) and true negative (TN). The TP is the number of correctly identified or classified as positive items. The FP is the number of incorrectly identified as positive items. The FN is the number of incorrectly identified as negative and TN is the number correctly identifies as negative items. From these measures, we have calculated the accuracy of the algorithm with the following equation:

(1) $$Accuracy=\frac{\mathrm{TP}+\mathrm{TN}}{\mathrm{TP}+\mathrm{FP}+\mathrm{TN}+\mathrm{FN}}$$

#### 4.3.2. Performance Analysis of Service Functions

**Figure 20:**
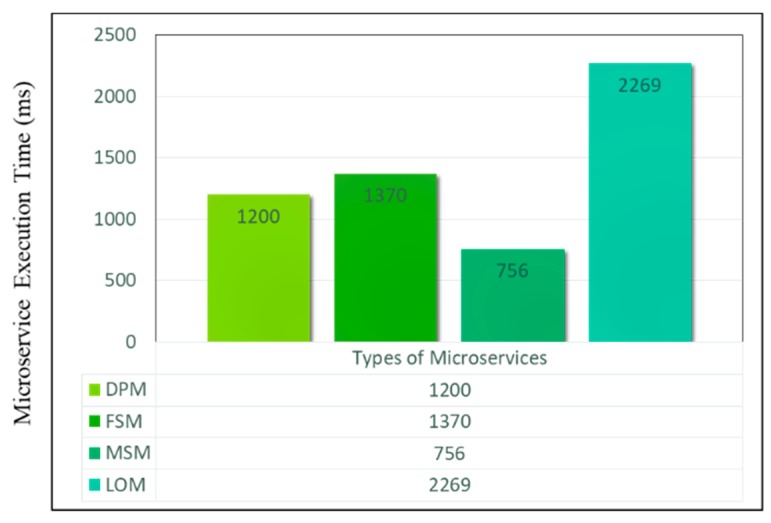
Execution time for four microservices.

On the other hand, the proposed system has also been analyzed from the microservices performance point of view. First, we discuss the performance analysis of microservices according to their functionalities. The graph in Figure 20 shows the microservice execution time with respect to selected services. These include Data Processing Microservice (DPM), Feature Selection Microservice (FSM), Model Selection Microservice (MSM) and Learning and Optimization Microservice (LOM). The data processing microservice performs imputation on missing values where its execution time shows the first run of this microservice. The FSM execution time is slightly different from the DPM and highly depends on the number of features, in this case, it uses 12 features from the selected dataset. The MSM takes very less time for the execution as compared to other microservices as it requires the selection of a model based on the characteristics of the data. Learning and Optimization function is the most time consuming as compared to all other microservice operations, this time varies based on the volume of the data selected for the learning model. In our case, the sample dataset requires less time for the first run of the LOM but as the number of samples increase, this increases the time as well.

**Figure 21:**
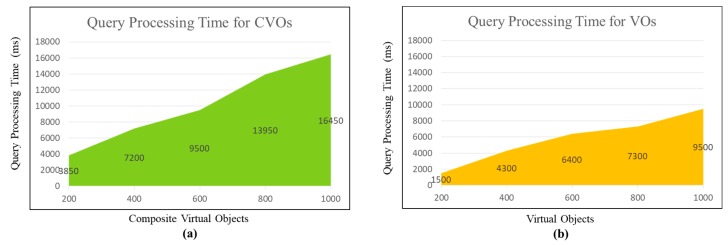
(**a**) Query processing time for CVOs. (**b**) Query processing time for VOs.

Secondly, we analyze the time required to query the virtual objects ontology. This experiment is achieved with an app in real-world settings. The app allows querying the objects with SPARQL query language. It is observed that the query processing time increases with the increasing number of CVOs (as shown in Figure 21a). Also, each CVO initiates a query to the SPARQL endpoint to read RDF triples, therefore increase in the number of CVOs also increases the overall query processing time respectively. On the other hand, the query processing time for VOs is observed to increase with respect to the increasing number of VOs. However, it is also seen that VOs query processing time is less than the CVOs (as depicted in Figure 21b). This is due to the fact that CVO triples additionally require the rules to be checked that apply on the VO data, which ultimately increases the overall query processing time.

## 5. Conclusions

Recently IoT analytics has gained popularity and spawned many opportunities based on the unleashed power of hidden patterns in the sensor data. However, several challenges still need to be resolved in order to utilize the true potential of IoT data analytics. These challenges include how to manage heterogeneous IoT data coming from diverse sources, how to efficiently analyze huge volumes of data with analytic techniques, how to develop modular services which enable the systems to scale well with analytic features, and how to make use of a large body of semantic web data in the data-driven pipeline. To deal with these challenges, this article has proposed a way to analyze data from heterogeneous sources and provided a multilayered model based on microservices and web objects concepts to support scalable and efficient services for IoT applications. The bottom layer provides the mechanisms to extract and process data from diverse sources with data-driven and knowledge-driven methods. The layer in the middle provides virtualization support to digitally represent the heterogeneous data. The top layer provides analytics microservices that enable IoT applications to extract hidden patterns from the data. A prototype was developed to analyze the feasibility of the model and a use case was demonstrated to perceive its applicability in real-world settings. In the future, we intend to evaluate the proposed model on other IoT use cases.

The proposed system has been analyzed from multiple views. In the first view, performance has been analyzed from the microservices point of view with respect to their analytic functionalities. It has been observed that among different microservices operations, the model selection takes very less execution time whereas learning and optimization function is the most time consuming as compared to other microservices. We have also analyzed the semantic processing features in our design, we compared the time required to query the virtual objects (VOs) and composite virtual objects (CVOs). The query processing time for both VOs and CVOs has been observed to increase with respect to the increase in their number. However, it has also been realized that VOs query processing time is less than the CVOs because the CVO triples require additional rule checking.

In the second view, we have used different machine learning techniques to evaluate the performance on two datasets to show a part of microservice operation performance. We have realized that model selection microservice will choose a model with the low error rate that can perform better in terms of accuracy. The selection of the model will affect the overall accuracy results in the analytic process.

Moreover, this article focused on predictive analytics with machine learning techniques, in future, we aim to use the complex event processing techniques to process real-time events from IoT devices and exploit the benefits of both techniques to enhance analytic processes.

## Figures and Tables

**Table 1:** PAMAP2 Dataset Attribute Description.

Attribute Description	Attribute ID	Attribute Description	Attribute ID
Time stamp	1	Chest IMU 3-D Acc (ms^−2^) ± 6 g	25–27
ID for the Activity	2	Chest IMU 3-D Gyr (rad/s)	28–30
Heart rate (beats per minute)	3	Chest IMU 3-D Mag (μT)	31–33
Temperature (Celsius) for IMU hand	4	Chest IMU 4-D orientation	34–37
Hand IMU 3-D Acc (ms^−2^) ± 16 g	5–7	Ankle Temperature (Celsius)	38
Hand IMU 3-D Acc (ms^−2^) ± 6 g	8–10	Ankle IMU 3-D Acc (ms^−2^) ± 16 g	39–41
Hand IMU 3-D Gyr (rad/s)	11–13	Ankle IMU 3-D Acc (ms^−2^) ± 6 g	42–44
Hand IMU 3-D Mag (μT)	14–16	Ankle IMU 3-D Gyr (rad/s)	45–47
Hand IMU 4-D orientation	17–20	Ankle IMU 3-D Mag (μT)	48–50
Chest IMU Temperature (Celsius)	21	Ankle IMU 4-D orientation	51–54
Chest IMU 3-D Acc (ms^−2^) ± 16 g	22–24		

**Table 2:** Notations used in the algorithm.

Notation	Description
⅀ф	Services objects where ф is a replacement for Mμ to denote microservices, Mo to denote virtual objects (CVOs and VOs).
μ₸	Represents all service objects returned in response to the query request.
μ	Represents the single instance iterated from the collection of Microservices.
M	Represents data model based on the specified ontology. Where ⅀Ms represents service data model, ⅀Mc represents context data model and user profile model is represented as ⅀Mu
qr	Q is the query to retrieve the available service templates.
qr’	Q’ is the query to retrieve the associated objects.
ℝ_e_	Entries of microservices in the registry.
Ώ	Set of objects’ that belong to the microservices category
ins Ώ	An instance of Ώ
xi	Iteration item of the list of microservice objects
λi	An instance of iteration items in the list of microservice objects
ℿ_matched_	List of all matched service items
ϖi	An instance of iteration items in the list of matched microservice items
ℿ_MRanked_	List of all matched ranking
Ɵ	Ranking value assigned to a service object
σj	Iterator item for the ranking list
ε	Threshold to rank a service object
Wf	Workflow for the composition of service objects
Ƥ	Priority queue to store ranked object instances.
Rth	Assigned Ranking

**Table 3:** Heart disease dataset description.

Attribute Information for the Cleveland Heart Disease Dataset		
1	Patient’s Age	Age of the patient in years
2	Sex	1 = Male; 0 = female
3	CP or Chest Pain Type	1 = typical angina; 2 = atypical angina; 3 = non-anginal pain; 4 = asymptomatic
4	Resting blood pressure (trestbps)	The resting blood pressure measured in mm Hg on admission to hospital
5	Serum cholestoral (chol)	Patient’s serum cholestoral level measured in mg/dL
6	Fasting blood sugar (fbs)	Fasting blood sugar level if >120 mg/dL then 1 = true and 0 = no
7	Resting electrocardiographic results (restecg)	The Resting electrocardiographic results. It is 0 = normal; 1 = ST-T wave abnormality; 2 = left ventricular hypertrophy by the criteria of Estes.
8	Maximum heart rate (thalach)	The maximum heart rate achieved
9	Angina with exercise (exang)	The exercise caused due to angina where 1 = yes and 0 = no
10	ST depression due to exercise (oldpeak)	The ST depression induced by exercise relative to the rest condition
11	Slop representing peak exercise (slop)	The slope for peak exercise ST segment where 1 = upsloping; 2 = flat; 3 = down sloping
12	Number of major vessels (ca)	The number of major vessel from 0 to 3 that are flourosopy colored
13	Thal value	The thal value is 3 = normal; 6 = fixed defect; 7 = reversable defect.
14	Diagnosis of heart disease (angiographic disease status)	This is the predicted attribute where value 0 = (<50 percent narrowing diameter) and value 1 = (>50 percent narrowing diameter)

**Table 4:** Diabetes dataset description.

Attribute Information for the Diabetes Database		
1	Pregnancies	Pregnancy with respect to the number of times
2	Glucose Level	The Plasma glucose concentration a 2 h in an oral glucose tolerance test
3	Blood Pressure Measure	The diastolic blood pressure in (mm Hg)
4	Skin Thickness	The triceps skinfold thickness (mm)
5	Insulin measure	Two-hour serum insulin (mu U/mL)
6	BMI	The body mass index (weight /height) kg/m
7	Diabetes Pedigree Function	The diabetes pedigree function
8	Age	Age of patient in years
9	Class	The class category variable in 0 or 1
